# Impact of c-di-AMP Accumulation, L-cysteine, and Oxygen on Catalase Activity and Oxidative Stress Resistance of *Listeria monocytogenes* 10403S

**DOI:** 10.3390/microorganisms13061400

**Published:** 2025-06-16

**Authors:** Mahide Muge Yilmaz Topcam, Dimitrios P. Balagiannis, Kimon Andreas G. Karatzas

**Affiliations:** Department of Food and Nutritional Sciences, School of Chemistry, Food and Pharmacy, University of Reading, Reading RG6 6AD, UK; m.yilmaztopcam@pgr.reading.ac.uk (M.M.Y.T.); d.balagiannis@reading.ac.uk (D.P.B.)

**Keywords:** *Listeria monocytogenes*, second messenger, c-di-AMP accumulation, phosphodiesterase, oxidative stress resistance, catalase activity, cysteine

## Abstract

*Listeria monocytogenes* is a foodborne pathogen frequently exposed to oxidative stress in diverse environmental conditions. Cyclic di-AMP (c-di-AMP) is a second messenger that plays a key role in stress resistance. This study investigates the role of *pdeA* (degrades c-di-AMP) and how c-di-AMP accumulation affects catalase activity and oxidative stress response and gene expression. Survival and catalase activity assays were conducted under oxidative stress, and c-di-AMP levels were quantified in *L. monocytogenes* 10403S under aerobic, anaerobic, and L-cysteine-supplemented conditions. Δ*pdeA*, which accumulates c-di-AMP, exhibited greater sensitivity to oxidative stress (4.6 log reduction for the wild type (WT) vs 7.34 log reduction for Δ*pdeA* at 10 h) and lower catalase activity than the WT in the early stationary phase. However, in the late stationary phase, while the catalase activity levels of Δ*pdeA* remained stable (~6.33 cm foam height), it became resistant to oxidative stress (5.85 log reduction). These findings indicate that *pdeA* contributes to catalase activity in *L. monocytogenes*. Transcriptomic analysis revealed differential expression of pathways mainly including pentose phosphate pathway, carbon metabolism, O-antigen nucleotide sugar biosynthesis and ABC transporters in Δ*pdeA* compared to WT. Our transcriptomic data provided promising insights into the molecular mechanisms underlying c-di-AMP regulation, which may enhance stress resistance. Moreover, oxidative stress led to increased intracellular c-di-AMP levels. Under L-cysteine supplementation, catalase activity levels in WT were similar to Δ*pdeA* (~1.86 cm foam height for both), but the latter showed enhanced oxidative stress resistance and c-di-AMP levels. Anaerobic conditions also elevated c-di-AMP levels in WT and Δ*pdeA* but resulted in greater oxidative stress sensitivity. Understanding these regulatory mechanisms provides valuable insights into oxidative stress resistance, with potential implications for food safety and pathogen control.

## 1. Introduction

*Listeria monocytogenes* is a Gram-positive, pathogenic bacteria commonly found in the environment and known for its ability to survive under adverse conditions [1]. It is an intracellular pathogen that can enter and multiply within the cells of mammals and causes listeriosis, which mostly affects immunocompromised individuals, the elderly and pregnant women [2]. *L. monocytogenes* is often found contaminating food, which is a significant concern for the food industry. When ingested, it can cross the intestinal epithelial barrier, invade cells, reproduce inside them by using various virulence factors such as internalins and haemolysis, and subsequently localise itself in different parts of the body [3]. During infection or growth in the host, *L. monocytogenes* must endure several stressors, such as the acidic conditions of the stomach, bile in the gastrointestinal tract, and low-oxygen or oxygen-free conditions. Its ability to effectively respond to these challenges is crucial for its presence and survival [4].

During extracellular or intracellular growth, *L. monocytogenes* is exposed to oxidative stress that requires the development of response mechanisms to cope with the harmful effects of reactive oxygen species (ROS) [5]. Host cells combat *L. monocytogenes* by initiating a respiratory burst, which causes exposure of invaders to ROS, including hydrogen peroxide and superoxide [6]. Superoxide dismutase (SOD) converts anions to hydrogen peroxide, and catalase breaks them down into oxygen and water. Therefore, catalase and SOD collaborate to detoxify ROS [5]. In addition, *L. monocytogenes* encounters low-oxygen or anaerobic conditions in the environment, including soil, vacuum packaging, and the host intestine. It is known that anaerobic conditions are involved in the virulence of *L. monocytogenes* [7]. Anaerobic growth increased the resistance of highly virulent *L. monocytogenes* strains towards bile salt compared to aerobic growth [8]. Moreover, anaerobically cultivated *L. monocytogenes* cells were found to have a higher invasion potential [9] as these conditions are an important signal for the upregulation of internalins that mediate invasion and initiate the intracellular cycle [10]. During its passage through the gastrointestinal tract, *L. monocytogenes* faces oxidative stress due to the ROS produced by the host immune system [11].

Cyclic di-adenosine monophosphate (c-di-AMP), which is a critical bacterial signalling nucleotide, regulates bacterial growth and replication, maintains cell wall integrity, modulates responses to various stress conditions, and plays a role in cell physiology, such as in potassium transport, synthesis of fatty acids, and biofilm formation, all of which are important for a successful bacterial infection [12,13,14]. While diadenylate cyclases (DACs) are responsible for the production of c-di-AMP, phosphodiesterases (PDEs) degrade it. Although c-di-AMP plays a crucial role in bacterial growth and metabolism, its excessive production could be harmful to cellular functions. PDE proteins, which are encoded by *pdeA* and *pgpH* in *L. monocytogenes* [15], allow bacteria to detect increased levels of c-di-AMP and initiate its hydrolysis, thereby regulating its concentration within the bacterial cell to maintain an optimal level [12,14].

The intracellular signals play a role in multi-stress resistance mechanisms, including oxidative stress, in various bacteria [16,17]. Essentially, the presence of two distinct PDEs in *L. monocytogenes* with unique functions suggests a complex regulatory system for c-di-AMP levels [15,18]. Disruption in this system can result in imbalance and potential toxicity from excessive c-di-AMP might hinder the bacterium’s ability to cope with various stresses. This highlights the importance of precise c-di-AMP control for maintaining bacterial resilience [18]. Elevated or decreased concentrations of c-di-AMP due to a mutation in PDEs and DACs result in sensitivity to oxidative stress in some bacteria [19,20,21,22,23,24,25], and a similar effect might also be observed in *L. monocytogenes*.

Moreover, nitrogen sources, glutamate, and glutamine [26] affect the c-di-AMP levels in many microorganisms, including *S. aureus* [27] and *B. subtilis* [28]. Potassium ions, which are crucial for facilitating glutamate transport during acid stress [29,30], have been shown to increase intracellular c-di-AMP levels in *L. monocytogenes* [31]. In *L. monocytogenes*, cysteine is another nitrogen-containing metabolite essential for growth and redox balance. It can be synthesized from glutamate and glycine or acquired directly from the host cytosol [32].

Although the role of c-di-AMP in oxidative stress resistance in *L. monocytogenes* is not yet fully understood, further investigation is needed to clarify its specific impact. Therefore, this study aimed to investigate the role of *pdeA*, and c-di-AMP accumulation in oxidative stress resistance, particularly through its influence on catalase activity. Since catalase protects cells against reactive oxygen species and its activity significantly affects oxidative stress resistance [33,34], we assessed how *pdeA* and c-di-AMP affect catalase activity under both aerobic and anaerobic conditions. Moreover, given its metabolic link to glutamate and its role in oxidative stress resistance, L-cysteine was included to evaluate its effect on intracellular c-di-AMP levels.

## 2. Materials and Methods

### 2.1. Bacterial Strains and Growth Conditions

*L. monocytogenes* 10403S WT (wild type) and its isogenic Δ*pdeA* mutant were used throughout this study (Table 1; University of Reading, KAK Collection). Overnight cultures in 3 mL BHI were mixed with DMSO (Sigma-Aldrich, Dorset, UK) to a final concentration of 7% (*v*/*v*) and stored at −80 °C as stock cultures. Prior to the experiments, stock cultures were streaked onto brain heart infusion (BHI) agar (LAB M, Lancashire, UK) and incubated at 37 °C overnight. Three single colonies from this medium were transferred to 3 mL sterile BHI broth (LAB M, Lancashire, UK) and incubated overnight at 37 °C with shaking (120 rpm). Subsequently, 1% (*v*/*v*) of these cultures were inoculated to prepare the cultures that were used in the experiments. These cultures were prepared in 250 mL conical flasks for aerobic conditions or 50 mL Falcon tubes for anaerobic conditions (Whitley MG 1000 Anaerobic Workstation, Don Whitley Scientific). Twenty mL of BHI broth or Defined Media (DM; containing 0.82 mM L-cysteine) [35] was used for the inoculum and incubated overnight at 37 °C with shaking (120 rpm). To investigate the effect of L-cysteine, DM was supplemented to achieve a final concentration of 1.57 mM L-cysteine. This was accomplished by volumetrically adding an appropriate volume of a 10 μg/mL L-cysteine stock solution (Sigma-Aldrich, Dorset, UK).

96 well plates were used to determine the growth of WT and Δ*pdeA.* Plates were incubated at 37 °C for 24 h, and the cell turbidity was measured using a microtiter plate reader (FLUOstar Omega, Ortenberg, Germany) at an optical density of 620 nm (OD_620_) at every 2 h.

### 2.2. Survival in the Presence of Hydrogen Peroxide

Cells were grown until the stationary phase for either 10 or 18 h, depending on the conducted experiment. Subsequently, they were challenged with various concentrations of a 30% solution of H_2_O_2_ (Sigma-Aldrich, Gillingham, UK) between 1.5% (resulted in the survival of cells) and 5.5% (resulted in a total inhibition of cells). A final concentration of 5.25% H_2_O_2_ was used for 10- and 18-h aerobically grown cells. Samples were taken every 20 min for 60 min. Before assessing H_2_O_2_ survival, the concentrations of H_2_O_2_ were optimized that should be used with each of the strains and conditions to avoid rapid death or complete survival that would not allow comparison between the Δ*pdeA* mutant and its corresponding WT strains. During the experiments, cultures were kept at 37 °C. Before and after the addition of H_2_O_2_, samples were taken, and serial decimal dilutions were prepared in maximum recovery diluent (MRD; Oxoid, Basingstoke, UK) and spread on BHI agar plates that were incubated at 37 °C. Subsequently, CFUs were enumerated to assess the concentration of cells in the cultures at each time point.

The same experiments were also conducted to investigate the effect of L-cysteine and the presence of oxygen. A final concentration of 1.5% and 4.5% of H_2_O_2_ was used for cells grown under aerobic in DM and L-cysteine-supplemented DM, respectively. Under anaerobic conditions, while 1% was used in DM, 2% was used for BHI and L-cysteine-supplemented DM.

### 2.3. Catalase Activity Assay

The catalase activity of *L. monocytogenes* cultures was assessed using a methodology described previously [34] with minor modifications. Briefly, cells were grown as mentioned in Section 2.1, and 500 μL of culture was transferred to a glass test tube containing 100 μL of 1% (*v*/*v*) Triton X-100 (Sigma-Aldrich, Dorset, UK). 100 μL of H_2_O_2_ (30% *v*/*v*) was then added to each test tube. Samples were taken every 2 h during the 24 h period. Oxygen release, resulting from the enzymatic degradation of H_2_O_2_, was observed as foam formation. After 5 min, the height of the foam, indicating oxygen levels and catalase activity, was measured in cm using a ruler and photographic images were taken.

### 2.4. Measurement of Intracellular c-di-AMP Levels

Intracellular c-di-AMP concentrations were measured with the method published by Oppenheimer-Shaanan (2011) with some minor changes [38]. A volume of 20 mL of cell culture was grown for 10 or 18 h. For aerobic conditions, the cells were grown in 250 mL flasks by shaking at 120 rpm, while under anaerobic conditions, they were grown in 50 mL Falcon tubes placed into a Whitley MG 1000 Anaerobic Workstation (Don Whitley Scientific) at 37 °C. Then, cultures were centrifuged at 9000 rpm for 10 min. The cell pellet was resuspended in 10 mL lysis buffer (10 mM Tris pH 8, 10 mM MgCl2 and 0.5 mg/mL lysozyme) and incubated at 37 °C for 30 min. Extracts were centrifuged at 9000 rpm for 10 min, and the supernatant was transferred to a clean tube. The extraction procedure was repeated twice by resuspending the pellets in 2 mL lysis buffer and collecting the supernatant. The collected supernatant was heated at 100 °C for 3 min and then supplemented with 100% ethanol to a final concentration of 70%. The supernatant was incubated for 20 min on ice and centrifuged at 7000 rpm for 10 min at 4 °C to separate insoluble material. It was then incubated at 80 °C for 1 h, and samples were air-dried at room temperature. Pellets of dried samples were resuspended in 1 mL of 1% formic acid, filtered (0.22 mm) and analysed in LC-MS.

An aliquot of the supernatant was filtered through a 0.2-µm nylon syringe filter (Fisher Scientific, Loughborough, UK) into an autosampler vial. An external calibration curve of c-di-AMP was built from 0.01 to 5 µg/L. Chromatographic separation was performed on an Agilent 1200 high-performance liquid chromatography (HPLC) system coupled to an Agilent 6410 triple quadrupole mass spectrometer equipped with an electrospray ionisation (ESI) source operating in positive mode. Separation was achieved using an Agilent Zorbax SB-C18 analytical column (2.1 × 100 mm, 1.8 µm) coupled to a Zorbax SB-C18 guard column (2.1 × 15 mm, 1.8 µm). The column temperature was maintained at 40 °C. The mobile phases consisted of Eluent A (water with 0.1% formic acid) and Eluent B (acetonitrile with 0.1% formic acid). The gradient elution program was as follows: 0–2 min, 100% A; 2–5 min, linear gradient to 100% B; 5–9 min, 100% B; 9–12 min, linear gradient to 100% A; followed by a 10-min post-run for column re-equilibration. The flow rate was set to 0.2 mL/min, and the injection volume was 5 μL. The ESI source parameters were as follows: gas temperature 350 °C, gas flow 10 L/min, nebuliser pressure 50 psi, and capillary voltage 4000 V.

To investigate the effect of H_2_O_2_ stresses on the c-di-AMP production/accumulation, the same protocol was also applied to the samples before the stress, just after the stress application (100 µL of 30% H_2_O_2_ sublethal dose), and 1 h after the stress application.

### 2.5. RNA Sequencing (RNA-seq) Sample Preparation and Analysis of L. monocytogenes Strains

Two separate transcriptomic analyses were conducted. In the first, *L. monocytogenes* 10403S WT (control) was compared to its isogenic Δ*pdeA* mutant to identify the impact of the loss of this gene in the overall transcription. In the 2nd transcriptomic analysis, *L. monocytogenes* 10403S WT in DM (control) was compared to WT in DM supplemented with L-cysteine (1.57 mM final concentration).

Overnight cultures grown in 3 mL BHI were inoculated (1% *v*/*v*) in 20 mL BHI in 250 mL conical flasks. Cultures were incubated at 37 °C for 10 h with 120 rpm. After growth, 16.66 mL of culture was mixed with 3.33 mL of phenol/ethanol (5/95%) solution. Cell suspensions were centrifuged at 5000× *g* for 10 min at 4 °C. The supernatant of the suspensions was discarded, and the cell pellets were frozen at −80 °C until further processing. RNA was isolated with RNeasy Mini Kit (Quiagen, Manchester, UK), and possible contamination with genomic DNA was removed with the use of Turbo DNA-free™ Kit (Quiagen, Manchester, UK). RNA quality and purity were assessed using the NanoPhotometerR spectrophotometer (IMPLEN, Westlake Village, CA, USA). Only samples that passed the quality control were processed further. RNA sequencing (RNA-Seq) and data analysis were performed by Novogene (Hong Kong, China).

A total amount of 3 µg RNA was used for RNA sample preparation. Sequencing libraries were constructed using NEBNext^®^ Ultra™ Directional RNA Library Prep Kit for Illumina^®^ (NEB, Ipswich, MA, USA), with index codes assigned to differentiate sequences for each sample. mRNA was purified from total RNA using poly-T oligo-attached magnetic beads.

rRNA was removed through a specialised kit and fragmented using divalent cations at elevated temperature in NEBNext First Strand Synthesis Reaction Buffer (5X). First-strand cDNA was synthesised by using a random hexamer primer and M-MuLV Reverse Transcriptase (RNaseH). Subsequently, second-strand cDNA synthesis was performed by using DNA Polymerase I and RNase H. During this step, dNTPs with dTTP were substituted with dUTP. The remaining overhangs were converted into blunt ends by exonuclease/polymerase. After adenylation of 3′ ends of DNA fragments, NEBNext Adaptor with hairpin loop structure was ligated to prepare for hybridisation. To select cDNA fragments of preferentially 150~200 bp in length, the library fragments were purified with AMPure XP system (Beckman Coulter, Beverly, MA, USA). Following this, 3 µL USER Enzyme (NEB USA) was added to size-selected and adaptor-ligated cDNA at 37 °C for 15 min, followed by 5 min at 95 °C. Polymerase chain reaction (PCR) was performed with Phusion High-Fidelity DNA polymerase, Universal PCR primers, and Index Primer. Lastly, the amplified library products were purified (AMPure XP system), and library quality was assessed on the Agilent Bioanalyzer 2100 system.

### 2.6. Statistical Analysis

All experiments were conducted in triplicate, and results were assessed using paired Student’s *t*-test and Tukey on Minitab Statistical Software, Version 22 (Minitab, LLC., State College, PA, USA, 2021). A *p*-value lower than 0.05 denotes statistically significant results, which are indicated by an asterisk (*) in figures or by different letters [39].

Differential gene expression analysis, including 3 replicates, was conducted on WT and Δ*pdeA* cells grown in BHI under anaerobic conditions to assess the impact of c-di-AMP accumulation on the expression of *L. monocytogenes* genes. The analysis was performed using the DESeq R package (1.18.0), which applies statistical methods for identifying differential expression in digital gene expression data based on a negative binomial distribution model. The resulting *p*-values were adjusted using the Benjamini and Hochberg method, and these are referred to as *padj* in this study. Genes with an adjusted *p*-value of less than 0.05 were classified as differentially expressed.

## 3. Results

### 3.1. Catalase Activity Levels During Growth

**Figure 1:**
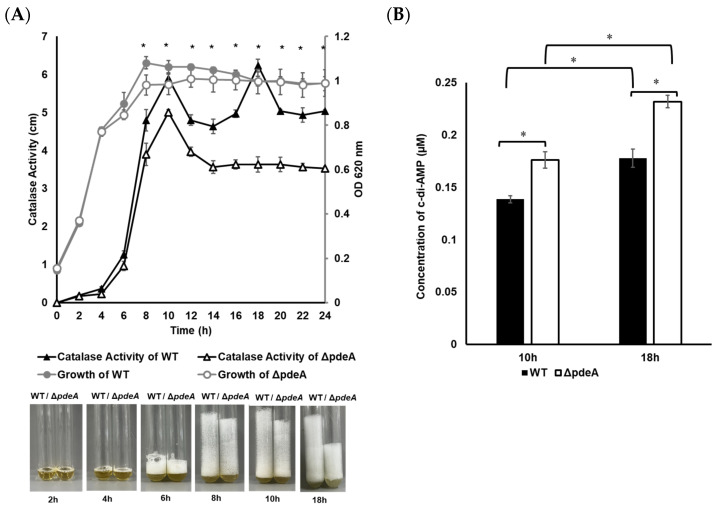
10403S WT (grey line with grey round markers) and Δ*pdeA* (grey line with white round markers) growth profiles at 37 °C under aerobic conditions with optical density was measured at 620 nm (OD_620_) every 2 h (**A**). Catalase activity of 10403S WT (black line with black triangle markers) and Δ*pdeA* cells (black line with white triangle markers) was measured every 2 h as cm of foam following catalase reaction, with photos taken (photos represent one of the replicates) (**A**). At the maximum catalase levels of WT cells (10 and 18 h), intracellular c-di-AMP concentrations of both WT (black bars) and Δ*pdeA* cells (white bars) were measured in triplicate (**B**). Error bars represent standard deviation, and asterisks show statistically significant differences in the catalase activity and c-di-AMP levels of WT and Δ*pdeA* (*p* < 0.05).

The bacterial growth of 1043S WT and Δ*pdeA* was monitored by measuring the optical density at 620 nm (OD_620_). No significant difference was observed between the OD_620_ values obtained from WT and Δ*pdeA* in BHI (*p* > 0.05; Figure 1A). Catalase activity of WT and Δ*pdeA* was also monitored throughout the growth period (Figure 1A). In the exponential phase and following 2 and 4 h of growth, the catalase levels of WT and Δ*pdeA* were similar (*p* > 0.05). The first difference between the strains was recorded at 8 h of growth (*p* > 0.05; Figure 1A) when the catalase activity of Δ*pdeA* (3.9 cm foam height) became significantly lower than that of WT (4.8 cm foam height) (*p* < 0.05; Figure 1A). At the stationary phase, Δ*pdeA* showed consistently reduced catalase activity with an average foam level of 3.6 cm compared to WT with an average of 6.2 cm (*p* < 0.05; Figure 1A).

c-di-AMP levels were measured at 10 and 18 h, where WT and Δ*pdeA* showed different catalase activity patterns. During the stationary phase (from 10 to 18 h), the intracellular c-di-AMP concentrations rose from 0.138 to 0.177 µM in the WT cells and from 0.176 to 0.232 µM in the Δ*pdeA* cells (*p* < 0.05; Figure 1B). Despite this significant increase, the catalase activity of Δ*pdeA* did not change during the stationary phase (*p* > 0.05; Figure 1A).

### 3.2. Survival Against H_2_O_2_

**Figure 2:**
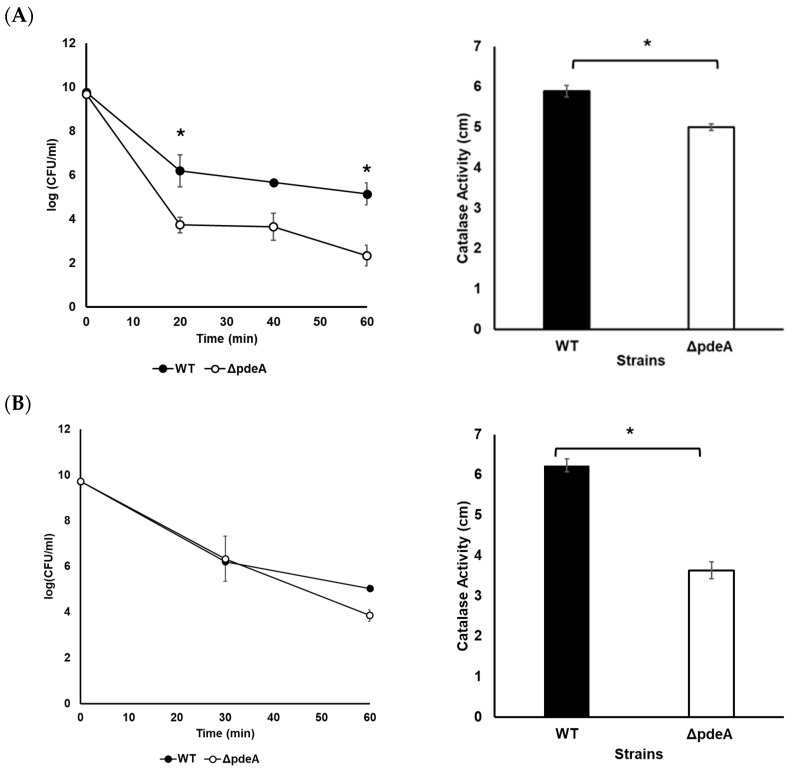
Survival and catalase activity of WT (black round markers and black bars, respectively) and Δ*pdeA* (white round markers and white bars, respectively) cultures grown for 10 h (**A**) and 18 h (**B**) following the addition of H_2_O_2_ to a final concentration of 5.25%. All the experiments were performed in three biological replicates. Error bars represent standard deviation, while asterisks denote statistically significant differences between the WT and Δ*pdeA* (*p* < 0.05).

A survival experiment was conducted, where a significant difference was seen in catalase activity levels (Figure 2). WT showed a 4.6 log reduction, while Δ*pdeA* showed a 7.34 log reduction following growth for 10 h, proving WTs were more resistant to oxidative stress than Δ*pdeA* (*p* < 0.05; Figure 2A). However, when cells grown for 18 h were challenged with 5.25% H_2_O_2_, an average of 4.65 and 5.85 log reduction was observed for WT and Δ*pdeA*, respectively, which showed a similar inactivation (*p* > 0.05; Figure 2B). The log reduction of WT at 10 and 18 h was not statistically significant (*p* > 0.05), whereas the log reduction of Δ*pdeA* showed a statistically significant difference between these time points (*p* < 0.05; Figure 2A,B).

### 3.3. Intracellular c-di-AMP Concentrations During the H_2_O_2_ Treatment

**Figure 3:**
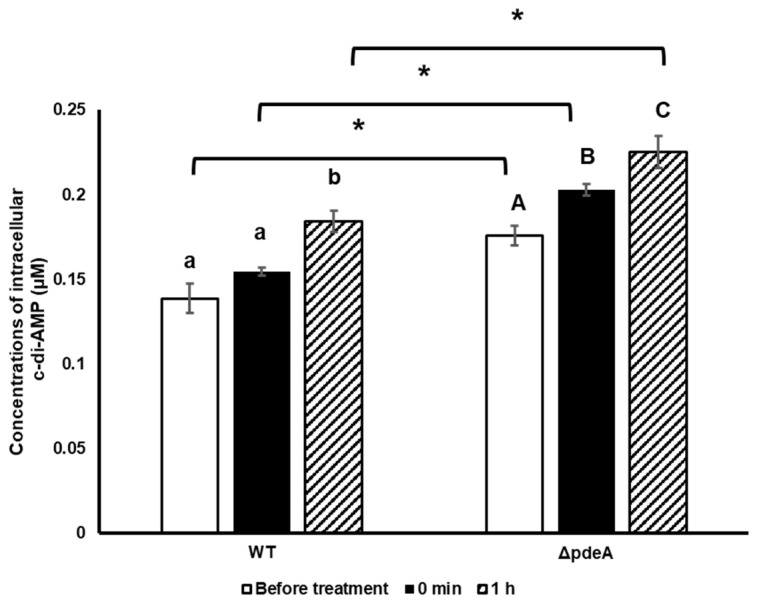
Intracellular c-di-AMP concentrations of 10hgrown WT and Δ*pdeA* cells prior to H_2_O_2_ stress (white bars), right after the H_2_O_2_ treatment (0 min; black bars) and after 60 min (1h; patterned bars). The average accumulation of the cells during the oxidative shock was measured from triplicate experiments. Error bars represent the standard deviation of three experiments. Lowercase letters indicate the difference between WT cells, while uppercase letters indicate the difference between Δ*pdeA* cells in BHI. Asterisks show significant differences between the strains (*p* < 0.05).

The levels of intracellular c-di-AMP were measured during the oxidative stress to assess the survival difference between 10-h-grown WT and Δ*pdeA*. Samples were taken before the H_2_O_2_ treatment, right after the H_2_O_2_ treatment, and after 1 h. Δ*pdeA* cells had higher intracellular c-di-AMP concentrations than WT cells, with an average concentration of 0.138 µM and 0.175 µM, respectively, before the H_2_O_2_ treatment (*p* < 0.05). Right after the H_2_O_2_ treatment, these concentrations were increased to 0.154 µM (*p* > 0.05) and 0.203 µM (*p* < 0.05). Subsequently, after 60 min H_2_O_2_ treatment, levels continued to increase and reached 0.184 µM (*p* < 0.05) and 0.225 µM (*p* < 0.05; Figure 3).

### 3.4. Transcriptomic Analysis Before the Stress Condition

**Figure 4:**
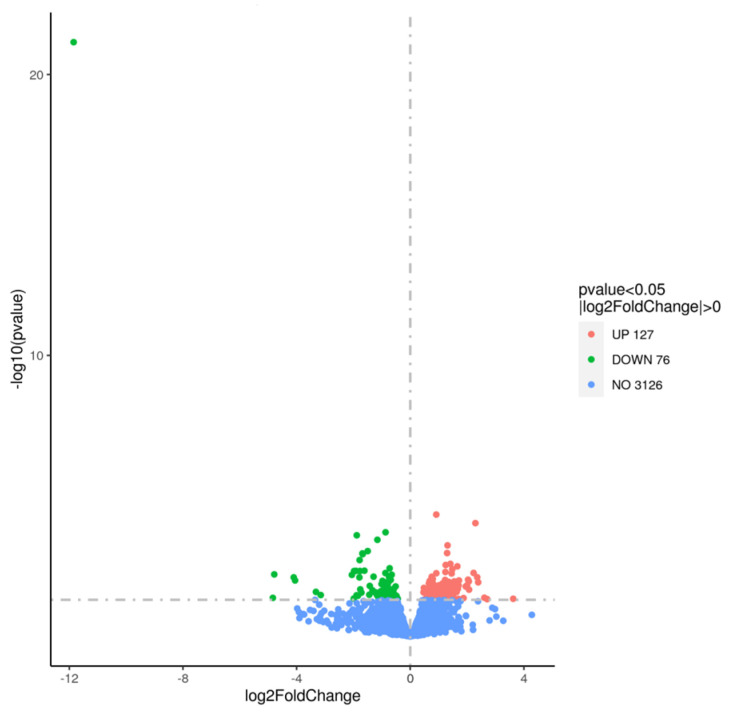
Volcano plot showing differential gene expression between Δ*pdeA* (treatment) and WT (control) strains grown for 10 h under aerobic conditions. In the volcano plot, the x-axis represents the log_2_-fold change in gene expression, while the y-axis represents the negative logarithm of the *p*-value (−log(*p*-value)), highlighting statistical significance. Genes with a log_2_-fold change greater than 0 and *p*-value < 0.05 are considered upregulated (coloured in red), while genes with a log_2_-fold change less than 0 are downregulated (coloured in green). Genes with no significant change are coloured in blue. The plot visually identifies differentially expressed genes between the two conditions.

Due to the difference in the survival of 10 h-grown WT and Δ*pdeA* cells, a full transcriptomic analysis of *L. monocytogenes* 10403S WT and Δ*pdeA* was conducted prior to the application of oxidative stress treatment. This comparison aimed to investigate the effect of different c-di-AMP levels between WT and Δ*pdeA* (as shown in Figure 1B). The summary of the transcriptome assembly statistics is shown in the supplementary data (Table S1). The error rate of a single base location sequencing was less than 1% in all groups. The Q2 and Q3 were equal to or higher than 97% or 93%, respectively. Transcriptomic data revealed that 127 genes were upregulated and 76 downregulated in the Δ*pdeA* compared to the WT (Figure 4).

**Figure 5:**
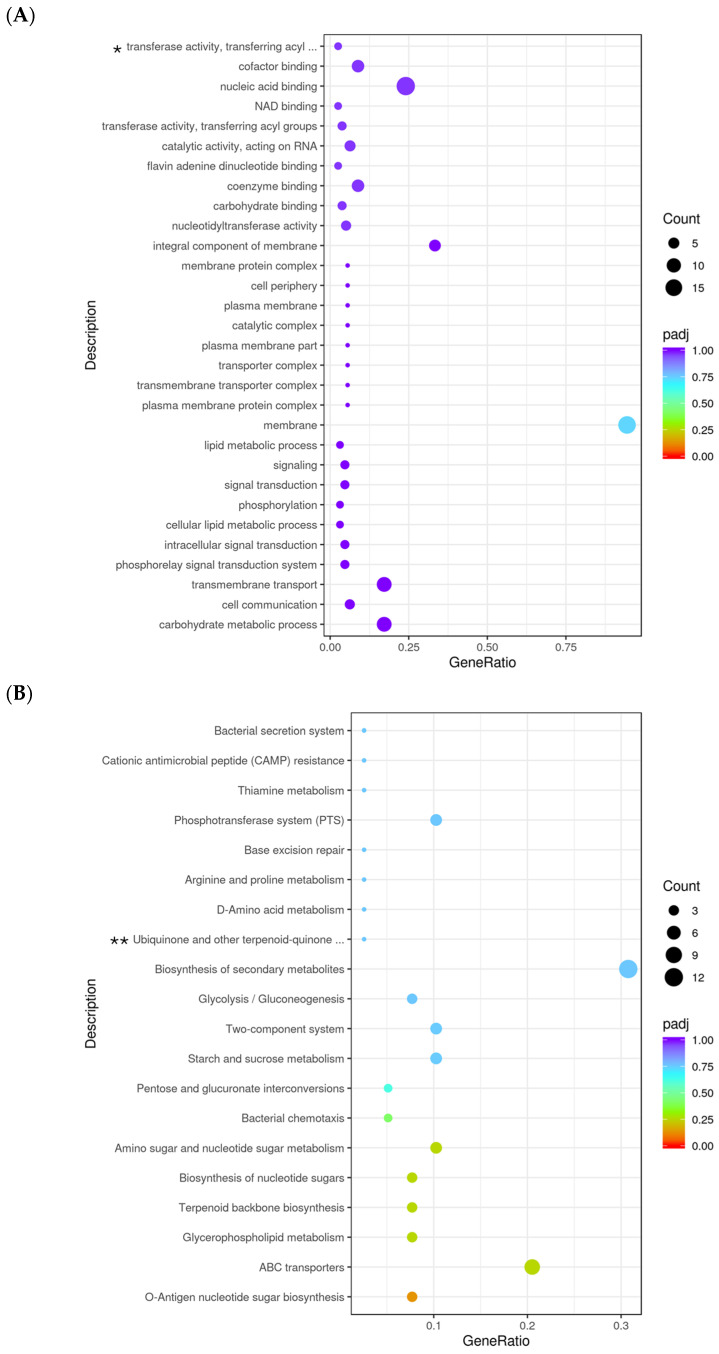

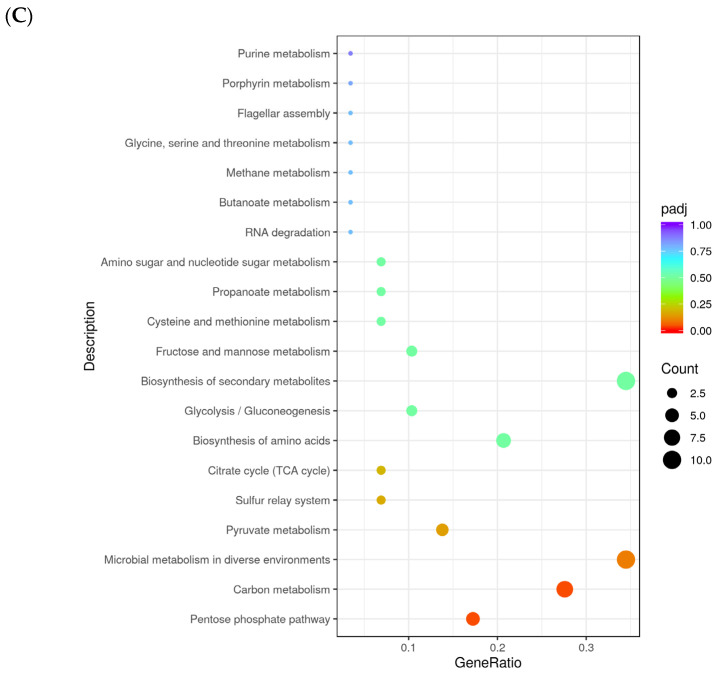
Gene ontology (GO) analysis with the most significant enrichment (**A**) and the most abundant pathways (KEGG); upregulated (**B**) and downregulated (**C**) of differentially expressed genes (DEGs) in Δ*pdeA* (treatment) and WT (control) strains grown for 10 h under aerobic conditions. The size of the dots is proportional to the number of genes; the closer the q value is to 0, the greater the extent of enrichment (* GO:0016747; ** KEGGID:lmt00130). KEGG pathway enrichment analysis identified several significantly enriched pathways among the differentially expressed genes (*padj* > 0.05; (**B**,**C**)). The most enriched pathways included “O-antigen nucleotide sugar biosynthesis” and “ABC transporters” with high gene ratios and low adjusted *p*-values. Other significantly enriched metabolic pathways included “glycerophospholipid metabolism”, “terpenoid backbone biosynthesis” and “biosynthesis of nucleotide sugar metabolism” (**B**). On the other hand, the downregulated gene set showed enrichment in core metabolic pathways, including “pentose phosphate pathway (PPP)” (*padj* < 0.05), “carbon metabolism” (*padj* < 0.05), “microbial metabolism in diverse environments”, “pyruvate metabolism” “sulfur relay system” and “citrate cycle (TCA cycle; Tricarboxylic Acid Cycle)”, with high gene ratios and strong statistical significance (*padj* < 0.5; (**C**)).

Gene Ontology (GO) enrichment analysis was performed to identify significantly enriched biological processes (BP), cellular components (CC), and molecular functions (MF) among differentially expressed genes in 10 h-grown Δ*pdeA* vs WT in BHI. The most significantly enriched GO term was “carbohydrate metabolic process”, with a high gene ratio and a low adjusted *p*-value, followed by “cell communication”, “transmembrane transport” and “signal transduction systems” (Figure 5A). A strong enrichment in membrane-associated functions was observed. Notably, the term “membrane” displayed the highest gene ratio (~0.75; Figure 5A), with a significantly low adjusted *p*-value (Figure 5A). Additionally, molecular function categories, including “nucleic acid binding” “NAD binding” and “cofactor binding” appeared enriched but were not statistically significant (*padj* > 0.05; Figure 5A).

Moreover, the genes involved in oxidative stress resistance, such as redox homeostasis [40], glutathione biosynthesis and transport [41] and cold shock proteins [42] were also assessed. There was no statistically significant difference in the transcription of these genes in Δ*pdeA* compared to the WT (*padj* > 0.05; Table S2).

### 3.5. The Effect of L-cysteine on the Catalase Activity and Survival of WT and ΔpdeA Under Aerobic Conditions

**Figure 6:**
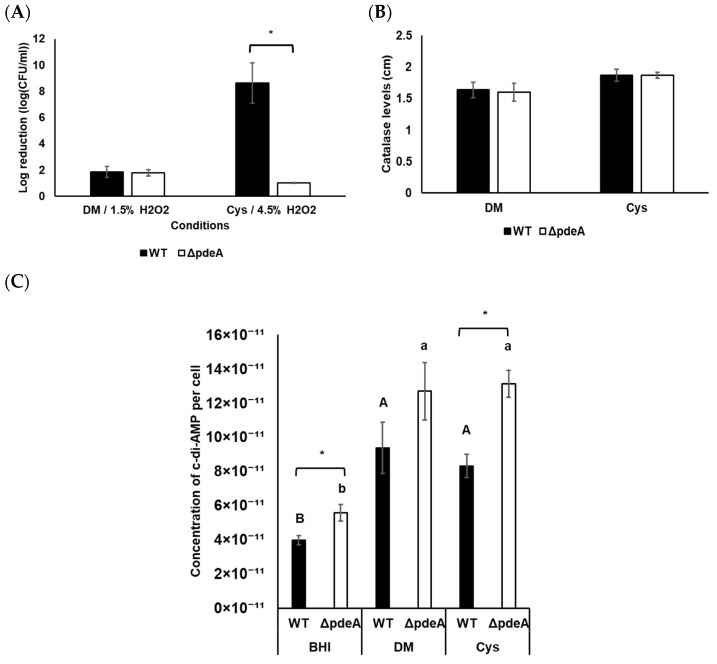
Oxidative stress survival (**A**), catalase activity levels (**B**) and intracellular c-di-AMP levels per cell (**C**) of 18 h aerobically grown WT and Δ*pdeA* cells. 1.5% and 4.5% H_2_O_2_ were applied for survival experiments to the cells grown in DM and L–cysteine–supplemented DM, respectively. Black bars represent WT, while white bars represent Δ*pdeA* cells. All the experiments were performed in triplicate, and average measurements are presented. Error bars represent standard deviation. Uppercase letters indicate the difference between WT cells, while lowercase letters indicate the difference between Δ*pdeA* cells in various media. Asterisks demonstrate statistical differences between the strains (*p* < 0.05).

In this part, in contrast to the previous experiment, DM was used for a clearer analysis of L-cysteine’s role without interference from other complex components present in BHI. WT and Δ*pdeA* cells showed an average of 1.84 and 1.77 log reduction in DM, respectively (*p* > 0.05; Figure 6A). Moreover, when 1.5% H_2_O_2_ was applied, both L-cysteine-supplemented WT and Δ*pdeA* were able to survive for 60 min. Therefore, 4.5% of H_2_O_2_ was applied to the cells grown in these conditions. L-cysteine supplementation led to a significantly greater resistance to oxidative stress in Δ*pdeA* compared to the WT, with a 7.6 log reduction difference (*p* < 0.05; Figure 6A). There was no significant difference in catalase activity levels between stationary phase cells of WT and Δ*pdeA* grown in DM (*p* > 0.05; Figure 6B). Similarly, L-cysteine supplementation did not affect the difference between the catalase activity levels of WT and Δ*pdeA* (*p* > 0.05; Figure 6B).

### 3.6. Intracellular c-di-AMP Concentrations Under Aerobic Conditions

Intracellular c-di-AMP levels in WT and Δ*pdeA* grown in BHI, DM, and L-cysteine-supplemented DM were measured under aerobic growth conditions. The concentration of c-di-AMP in Δ*pdeA* was higher than in WT cells (*p* < 0.05; Figure 6C). The aerobically-grown cells in BHI had the lowest concentration of c-di-AMP, with an average of 3.96 × 10^−11^ and 5.57 × 10^−11^ µM/CFU for WT and Δ*pdeA* respectively (*p* < 0.05; Figure 6C). However, growth in DM resulted in higher c-di-AMP concentrations than BHI (*p* < 0.05). Additionally, it was observed that L-cysteine supplementation did not change c-di-AMP concentrations compared to DM under aerobic conditions (*p* > 0.05; Figure 6C). Yet, Δ*pdeA* had higher c-di-AMP concentrations than the WT with L-cysteine supplementation (*p* < 0.05; Figure 6C).

### 3.7. Survival and Catalase Activity of Anaerobically Grown WT and ΔpdeA Cells

**Figure 7:**
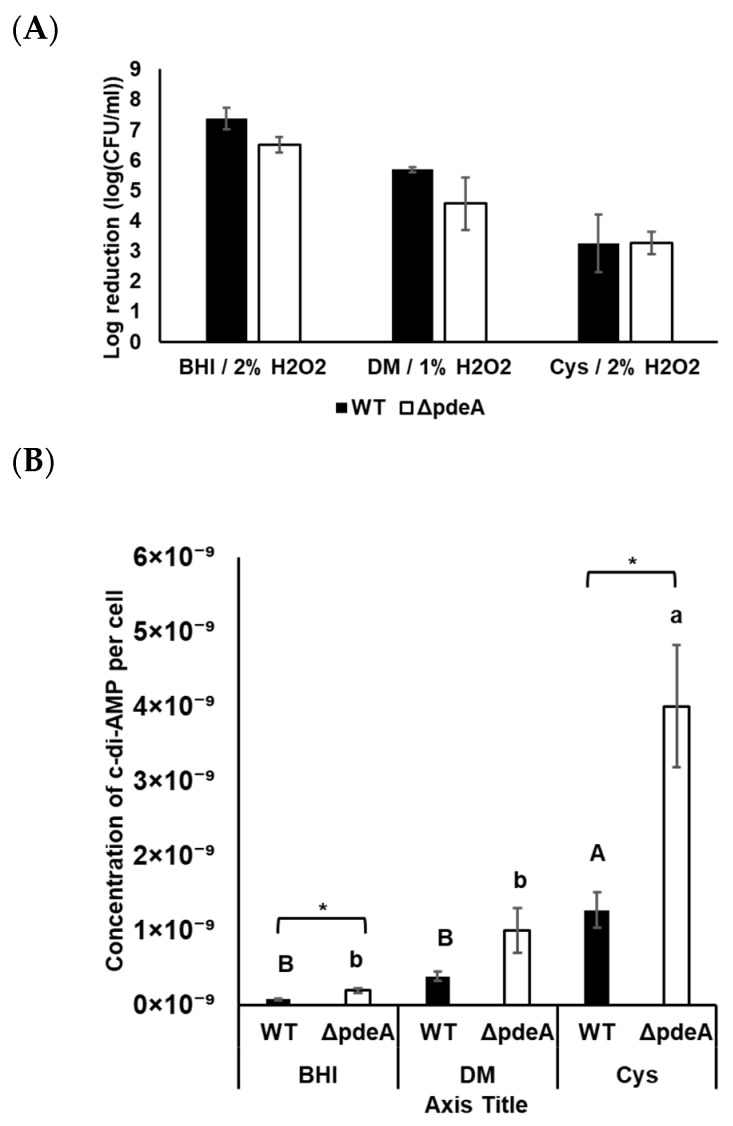
Survival of WT (black bars) and Δ*pdeA* (white bars) cultures grown until 18 h in BHI, DM and L-cysteine-supplemented DM under anaerobic conditions (**A**); and intracellular c-di-AMP levels of WT (black bars) and Δ*pdeA* (white bars) under the same growth conditions (**B**). 1% was used in DM, 2% was used for BHI and L-cysteine-supplemented DM. All the experiments were replicated three times, and average values are presented. Error bars represent standard deviation. Uppercase letters indicate the difference between WT cells, while lowercase letters indicate the difference between Δ*pdeA* cells in various media. Asterisks demonstrate statistical differences between the strains (*p* < 0.05).

Survival experiments were also conducted under anaerobic conditions. It is known that early stationary phase cells under anaerobic conditions can be more susceptible to oxidative stress [43,44]. Therefore, we only tested 18-h-grown cells under anaerobic conditions. Both strains were more sensitive towards oxidative stress compared to aerobic growth conditions (Figure 7A). Although 5.25% of H_2_O_2_ was applied to the aerobically grown cells in BHI, a maximum of 2% of H_2_O_2_ provided a clear survival curve for the anaerobically grown cells. In BHI, WT and Δ*pdeA* cells showed an average of 1.78 and 2.72 log reduction, respectively (*p* > 0.05; Figure 7A). However, there was no significant difference between WT and Δ*pdeA,* neither in DM nor in DM supplemented with L-cysteine (*p* > 0.05; Figure 7A). Furthermore, the catalase activity of WT cells was at immeasurable levels in BHI, DM, and L-cysteine-supplemented DM. Even though the sample volume was increased, no measurable levels of catalase activity were obtained from either WT or Δ*pdeA*.

### 3.8. Intracellular c-di-AMP Concentrations Under Anaerobic Conditions

c-di-AMP in WT and Δ*pdeA* grown in BHI, DM, and L-cysteine-supplemented DM were measured under anaerobic conditions. Under anaerobic conditions, c-di-AMP levels in Δ*pdeA* were significantly higher than in WT (*p* < 0.05; Figure 7B). Additionally, compared to the aerobic conditions, intracellular c-di-AMP concentrations per cell were higher in both strains grown under anaerobic conditions, regardless of the media used (Figure 6C and Figure 7B).

Similarly to aerobic conditions, c-di-AMP levels in BHI were measured lower compared to those in DM and L-cysteine-supplemented DM (*p <* 0.05; Figure 7B). In contrast, L-cysteine supplementation resulted in significantly higher c-di-AMP concentrations under anaerobic conditions, with 1.26 × 10^−9^ and 3.99 × 10^−9^ µM/CFU µM in WT and Δ*pdeA* respectively (Figure 7B). The difference in the c-di-AMP concentrations between anaerobically grown WT and Δ*pdeA* in L-cysteine-supplemented DM was higher compared to those grown in BHI and DM (*p* < 0.05; Figure 7B).

### 3.9. Transcriptomic Data Showing the Effect of L-cysteine Supplementation on c-di-AMP Homeostasis Genes

We showed that L-cysteine supplementation increases c-di-AMP levels in both WT and Δ*pdeA* cells under anaerobic conditions (Figure 7B). To better understand the broader effects of L-cysteine, we conducted a complete transcriptomic analysis under anaerobic conditions. While some parts of the transcriptomic data are reported in another study (Yilmaz Topcam & Karatzas, unpublished data) [45], the current analysis focuses on c-di-AMP DACs and PDEs of the transcriptome that were not addressed earlier. The transcriptomic data obtained from overnight cultures of *L. monocytogenes* 10403S WT cells in DM (control) and DM supplemented with 1.57 mM L-cysteine (treatment) showed that L-cysteine supplementation significantly downregulated the c-di-AMP PDEs (*padj* < 0.05; Table 2).

## 4. Discussion

Second messenger c-di-AMP is known to influence a range of physiological processes, including growth, cell wall homeostasis, potassium ion transport, DNA integrity, fatty acid synthesis and biofilm formation, all of which contribute to bacterial infection [12,13,14]. It is also implicated in multi-stress resistance pathways, which include oxidative stress resistance and heat shock responses [16,17]. Although the reaction of different microorganisms towards oxidative stress and the role of c-di-AMP have been investigated previously [19,20,21,22,46], the precise role of c-di-AMP on the oxidative stress resistance of *L. monocytogenes* remains unclear. Moreover, previous studies have not considered the role of catalase activity, which is required for defence against oxidative stress [34]. Therefore, for the first time, we investigated the role of *pdeA* and potentially that of c-di-AMP on the catalase activity of *L. monocytogenes* 10403S.

Catalase activity levels of both exponential-phase WT and Δ*pdeA* cells were lower than those at the stationary phase (Figure 1A), which is consistent with our previous results [44]. While the catalase activity of both strains reached the peak value at 10 h of growth, another peak was observed at 18 h in WT (Figure 1A). Similar results to our findings for WT were also observed previously for *Enterobacter aerogenes*, with two separate peak points in the catalase activity in the stationary phase during the growth [47]. This fluctuation in the catalase activity of the WT observed across different growth phases may reflect regulatory mechanisms beyond the transcription of only catalase gene present in *L. monocytogenes* (*kat*). Indeed, oxidative stress resistance in *L. monocytogenes*, particularly during different growth stages, might be affected by some factors such as mRNA stability, translation efficiency, or post-translational modifications of antioxidant enzymes [44]. In contrast to the increased catalase activity of WT, the catalase activity of Δ*pdeA* remained stable through the stationary phase, indicating the impact of PdeA on catalase activity in WT. Moreover, an increased concentration of c-di-AMP was recorded in both WT and Δ*pdeA* from 10 to 18 h (Figure 1B), where the cell number of the strains did not show a significant change (Figure 1A and Figure 2; initial counts). This suggests that the maintenance of the c-di-AMP homeostasis is important for the catalase activity of *L. monocytogenes* 10403S in the stationary phase, however, high concentrations of c-di-AMP as a result of its accumulation do not appear to further enhance catalase activity.

In the present study, we show that the Δ*pdeA* exhibited an increased oxidative stress resistance at 18h (Figure 2). This increased resistance of Δ*pdeA* may be associated with the c-di-AMP levels (Figure 1B), which could enhance survival by promoting improved DNA repair [21], despite stable catalase activity during the stationary phase (Figure 1A). In various bacteria, low levels of c-di-AMP (due to the over-expression of PDEs) impair DNA repair, whereas high levels (due to the mutation in PDEs) promote cell survival towards DNA damaging agents (such as H_2_O_2_, methyl methane sulfonate, day-night cycle, and UV) [19,20,21,22]. While c-di-AMP has been implicated in the DNA repair process, its specific role in oxidative stress resistance in *L. monocytogenes* remains unclear and requires more investigation.

Regarding the survival experiments, intracellular c-di-AMP levels were also measured following H_2_O_2_ treatment. It is worth mentioning that a sublethal dose of H_2_O_2_ which did not affect cell counts was applied to the cells. Oxidative stress treatment resulted in continuously increased intracellular c-di-AMP levels in both WT and Δ*pdeA* (*p* < 0.05, Figure 3). Thus, here we show for the first time that oxidative stress induces a continuous increase in c-di-AMP concentrations in response to oxidative stress in *L. monocytogenes* (Figure 3; WT cells). Contrary to our results, sporulating *B. subtilis* cells reacted to oxidative stress by decreasing c-di-AMP concentrations [38]. In *L. monocytogenes,* the levels of (p)ppGpp, another secondary messenger that regulates the stringent response through various interactions, are controlled by the RelA/SpoT homolog and CbpB. CbpB activates RelA, leading to (p)ppGpp synthesis, while c-di-AMP inhibits this activation [48]. The role of (p)ppGpp on starvation, antibiotic resistance, and osmotic stress has been elucidated [49]. Also, it is known that elevated (p)ppGpp levels under these stress conditions inhibit cyclic-di-AMP phosphodiesterase, resulting in increased c-di-AMP levels [50] and therefore *prfA* activation [51,52]. *lmo0779* (uncharacterized function), *cbpA* (a cyclic di-AMP binding protein [53]) and *ygbB* (2-C-methyl-D-erythritol2,4-cyclodiphosphate synthase) are important for oxidative stress resistance of *L. monocytogenes* when PrfA is activated [51]. MEP (methylerythritol phosphate) pathway, which plays a role in oxidative stress [54], uses pyruvate as a substrate and involves the *ygbB* gene [55]. On the other hand, the regulation of pyruvate entering the TCA cycle by inhibiting pyruvate carboxylase (*pycA*) is controlled by c-di-AMP signalling [37]. Elevated c-di-AMP levels in Δ*pdeA* and WT likely inhibited *pycA* activity and resulted in the interruption of pyruvate entrance to the TCA cycle. Thus, the increased concentration of c-di-AMP under oxidative stress (Figure 5) is likely due to disruption of the TCA cycle, the use of pyruvate by the MEP pathway, and due to an activated *ygbB.* Since (p)ppGpp has been shown to protect against oxidative stress in other microorganisms [56], our findings suggest a link between (p)ppGpp signalling and c-di-AMP levels in *L. monocytogenes* against oxidative stress resistance.

Due to the differences in oxidative stress resistance and catalase activity levels between WT and Δ*pdeA*, a whole transcriptomic analysis was conducted for the 10 h aerobically grown WT and Δ*pdeA* cells before oxidative stress. The enrichment of GO categories was observed, but the statistical significance was relatively modest (Figure 5A). This suggests that while biological processes related to membrane integrity, metabolism, and signalling were affected by the loss of *pdeA*, the magnitude of these changes at the global level was subtle. It is possible that the sensitivity of 10 h grown Δ*pdeA* to oxidative stress arises from cumulative minor disruptions across the multiple metabolic and membrane-associated pathways rather than one major factor alone.

KEGG pathway enrichment analysis of the downregulated genes revealed significant enrichment of key energy-related pathways, including the pentose phosphate pathway (PPP), carbon metabolism, pyruvate metabolism, and citrate cycle (TCA cycle) (Figure 5C). These pathways are the top significant energy pathways playing a role in oxidative stress [57,58,59,60]. Therefore, the downregulation of mainly these pathways might contribute to the sensitivity of 10 h grown Δ*pdeA* cells against oxidative stress. PPP has two parts, which are the oxidative branch and the non-oxidative branch. The oxidative branch helps break down glucose to produce NADPH [61], a crucial reducing agent that powers antioxidant systems like glutathione and thioredoxin [62]. Meanwhile, the non-oxidative branch generates five-carbon sugars needed for nucleotide synthesis, helping in the repair of DNA damage caused by ROS [61]. Earlier, it has been shown that the oxidative branch becomes especially active during sudden oxidative stress to provide more NADPH [63]. Additionally, in *L. monocytogenes,* the enzyme in the non-oxidative part of the PPP called ribulose-5-phosphate 3-epimerase (Rpe), converts ribulose-5-phosphate into glyceraldehyde-3-phosphate and fructose-6-phosphate. Both are part of the glycolysis pathway [64,65], which is also downregulated in Δ*pdeA* compared to WT (Figure 5C).

Oxidative stress affects the TCA cycle in microorganisms in different ways. In some cases, TCA cycle activity increases, with elevated levels of intermediates like fumarate, malate, α-ketoglutarate, and succinate, sometimes involving the GABA shunt for stress resistance [66]. Furthermore, the activity of the TCA cycle leads to ROS production [66,67], which triggers SOS response (bacterial DNA repair mechanism) to protect cells against ROS [57]. In other situations, it leads to the downregulation of the TCA cycle and glycolysis as part of an energy-saving survival strategy [66]. Despite these variations, many microbes commonly shift glucose metabolism towards the PPP to generate NADPH, which helps combat oxidative damage. Recent studies have also proven this by showing that the TCA cycle plays a limited role in *L. monocytogenes* [63] and *Escherichia coli* [67] oxidative stress response. It is obvious that elevated c-di-AMP levels in WT (Figure 3) would interrupt the TCA cycle. Overall, these support that downregulation of PPP is a key factor contributing to the sensitivity of 10 h grown Δ*pdeA* against oxidative stress in *L. monocytogenes*.

Moreover, Siletti et al. (2024) have found that c-di-AMP accumulation in *L. monocytogenes* disrupts glutathione metabolism, which might result in sensitivity to oxidative stress [41]. Our transcriptomic data showed that the expression of *gshAB*, which encodes glutathione biosynthesis, was not affected significantly by the accumulation of c-di-AMP at the end of 10 h (*padj* > 0.05; Table S2). However, pathways contributing to glutathione levels in the cell, including PPP (*padj* < 0.05), pyruvate metabolism, glycolysis/gluconeogenesis, and cysteine and methionine metabolism, were also downregulated (*padj* > 0.05; Figure 5B). Even though we confirmed the disruption in glutathione metabolism due to c-di-AMP accumulation (Yilmaz Topcam & Karatzas, unpublished data) [42], this phenomenon occurs at the late stationary phase. As previously mentioned, at 18 h, the accumulation of c-di-AMP enhanced cellular robustness against oxidative stress, which was unexpected considering the disruption of glutathione metabolism. This supports the idea that the increased resistance may involve additional mechanisms, rather than solely the disruption of glutathione metabolism.

Downregulation of genes mainly in the PPP, carbon metabolism, sulfur relay, pyruvate metabolism, glycolysis/gluconeogenesis, and cysteine and methionine metabolism system might lead to the sensitivity of 10 h grown Δ*pdeA* under oxidative stress [60,63,68]. Apart from the above pathways, we also investigated genes that are involved in oxidative stress resistance-related. There was no significant difference in the transcription of oxidative stress resistance genes (Table S2). Even though most of these genes, such as *ohrR*, *rex*, *prfA*, *kat* and *recA*, were downregulated with the c-di-AMP accumulation, the changes were not statistically significant (*padj* > 0.05). Superoxide dismutase protein *sodA* was slightly downregulated with the deletion of *pdeA* (−0.43 log_2_fold change, *padj* > 0.05). Previously, in *S. aureus*, the superoxide dismutase gene (*sodM*) was also shown to be downregulated in PDE mutants [20,46]. Similar to our findings at 18 h (comparison survival of Δ*pdeA* and WT), Corrigan et al. (2015) also did not observe an additional protection effect of c-di-AMP on survival under oxidative stress (H_2_O_2_ exposure for 30 min), even though a 5.56-fold downregulation of *sodM* was recorded [46]. We also showed that at 10 h of growth, *disA* and *cdaA*, which are responsible for the production of c-di-AMP and important for repairing DNA damage caused by alkyl groups and H_2_O_2_ [21], were insignificantly downregulated (−0.55 and −0.64 log_2_fold change respectively, *padj* > 0.05).

These downregulated pathways and gene expressions before the application of H_2_O_2_ may make the Δ*pdeA* cells already sensitive towards oxidative stress. Similar to our findings, the subtle effect of low levels of c-di-AMP has been recently observed by Tu et al. (2024). It has been found that at normal and low c-di-AMP levels, β-lactam antibiotic resistance diminishes, whereas, upon c-di-AMP accumulation, β-lactam antibiotic resistance increases [69]. Longer incubation periods might provide a clearer change in the expression levels of those genes, due to higher c-di-AMP accumulation in Δ*pdeA* cells. In fact, even if oxidative stress-related genes were significantly downregulated at a later time point, this alone would not explain the resistance of Δ*pdeA* observed in 18 h, as previously demonstrated by Gándara & Alonso (2015) [21]. Instead, the elevated resistance of Δ*pdeA* might be related to membrane-associated pathways and improved DNA repair due to high concentrations of c-di-AMP accumulation.

O-antigen nucleotide sugar biosynthesis, ABC transporters, glycerophospholipid metabolism, terpenoid backbone biosynthesis, biosynthesis of nucleotide sugars, amino sugar and nucleotide sugar metabolism, bacterial chemotaxis are the most significantly upregulated pathways, in aerobically 10 h grown Δ*pdeA* cells (vs. WT) (Figure 5B). O-antigen nucleotide sugar biosynthesis, nucleotide sugar metabolism, and glycerophospholipid metabolism all contribute to cell wall and membrane integrity [70,71,72] which are significant in dealing with oxidative stress resistance [73]. Moreover, MEP is one of the main routes for the terpenoid backbone biosynthesis that confirms the protectant role of this pathway to oxidative stress [74]. Further upregulation of these pathways in the following time points might contribute to enhanced oxidative stress resistance of Δ*pdeA*. Overall these support the notion that as c-di-AMP accumulates over time, its effect on biological and molecular functions, and cellular components, would become more pronounced.

Cysteine is essential for growth and redox balance. It can be synthesised from glutamate and glycine or acquired directly from the host cytosol [32]. Due to the effect of glutamate and glutamine on c-di-AMP concentrations in *S. aureus* and *B. subtilis* [27,28], and the downregulation of cysteine and methionine metabolism in Δ*pdeA* (Figure 5C), we also tested the impact of L-cysteine in *L. monocytogenes* in this study. There was no difference in catalase activity or survival under oxidative stress (Figure 6A,B) and intracellular c-di-AMP concentrations (Figure 6C) when the cells were grown in DM. As expected, supplementation with L-cysteine made 10403S WT cells more robust towards H_2_O_2_ treatment [54,75], which can be inferred from the applied H_2_O_2_ concentration for survival experiments (Figure 6A). On the other hand, catalase activity levels of both strains were not affected significantly compared to DM (*p* > 0.05) and were similar to each other with L-cysteine supplementation (*p > 0.05*; Figure 6B). The discrepancy between catalase activity levels and oxidative stress resistance under aerobic conditions raises the question of whether intracellular c-di-AMP concentrations might affect survival rather than catalase activity under these conditions. Therefore, the intracellular c-di-AMP concentrations of WT and Δ*pdeA* were measured. As expected, Δ*pdeA* cells accumulated more c-di-AMP than WT in BHI (*p* < 0.05; Figure 6C). Loss of *pdeA* did not result in the accumulation of considerable concentrations of c-di-AMP levels in DM (Figure 6C). This is likely because c-di-AMP is particularly required for metabolic adjustments in nutrient-rich environments and within host cells, but not in a minimal media [8]. On the other hand, supplementation of aerobically grown cells with L-cysteine did not affect intracellular c-di-AMP levels of either WT or Δ*pdeA*, but the difference between strains was found significant (*p* < 0.05; Figure 6C). The higher c-di-AMP concentrations in Δ*pdeA* might explain the survival against oxidative stress. However, similar catalase activity levels or c-di-AMP concentrations in DMs are likely because cysteine is oxidised to cystine in the presence of oxygen [76], preventing any observable effect of cysteine as a compound. To see the exact role of cysteine and know the effect of the presence of oxygen on c-di-AMP concentrations [27], the same experiments were also conducted under anaerobic conditions.

Under anaerobic growth, L-cysteine led to a significant increase in c-di-AMP levels of both WT and Δ*pdeA* (*p* < 0.05; Figure 7B). This observation suggests that L-cysteine may affect the production and degradation of c-di-AMP in *L. monocytogenes*. Transcriptomic data investigating the effect of L-cysteine supplementation in the WT (under anaerobic conditions) showed that the expression of *dacA* (responsible for c-di-AMP production in *L. monocytogenes*) resulted in a significant downregulation (*p-adj* < 0.05; Table 2). This downregulation is likely because of nitrogen-sensitive control of c-di-AMP synthesis, which was previously shown in *B. subtilis* in the presence of glutamine [28]. Moreover, Whiteley et al. (2015) found that *dacA* is not essential for growth in minimal media. This was associated with the requirement for glutamate, which is necessary for producing several metabolites under nutrient stress [8]. Cysteine is a key precursor for glutathione synthesis [32]. Therefore, it would decrease the requirement for glutamate and glutathione. L-cysteine supplementation may reduce demand for the other metabolic compounds in DM; consequently, it might decrease the need for *dacA*. Moreover, both PDEs, *pdeA* and *pgpH*, were significantly downregulated (*p-adj* < 0.05; Table 2) in response to L-cysteine supplementation, which might explain the elevated c-di-AMP concentrations of WT in L-cysteine-supplemented DM (Figure 7B).

We found that anaerobically grown *L. monocytogenes* WT and Δ*pdeA* cells were more sensitive to oxidative stress than aerobically grown cells (Figure 7A), and catalase activities were under the detectable limits, which are consistent with previous findings in the literature [77,78]. Whole transcriptomic data comparing aerobic and anaerobic growth of *L. monocytogenes* EGD performed by Müller-Herbst et al. (2014) showed that anaerobic incubation led to the downregulation of the *kat* gene [7], which can support our lower catalase activity results under anaerobic conditions. Surprisingly, while aerobically stationary phase grown Δ*pdeA* was more robust than WT against oxidative stress with L-cysteine supplementation, anaerobically grown Δ*pdeA* and WT had similar survival rates under oxidative stress.

Intracellular c-di-AMP concentrations of anaerobically grown Δ*pdeA* and WT were also measured. Similarly to aerobic conditions, the difference between Δ*pdeA* and WT grown in DM was not statistically different. This again supports the requirement of c-di-AMP in nutrient-rich environments for metabolic adjustments [8]. Moreover, the pattern in BHI was the same as under aerobic conditions (Figure 6C and Figure 7B). *L. monocytogenes* can produce ATP (Adenosine Triphosphate) through substrate-level phosphorylation or oxidative phosphorylation. Oxidative phosphorylation results in a higher ATP yield, which leads to better growth of *L. monocytogenes* EGD under aerobic conditions [7]. Importantly, since intracellular c-di-AMP levels were normalized per cell, the differences observed between aerobically and anaerobically grown cells reflect true intracellular changes rather than being a consequence of differences in growth. This suggests that oxygen availability directly influences c-di-AMP homeostasis in *L. monocytogenes*, independent of cell number.

On the other hand, Müller-Herbst et al. (2014) have demonstrated that the expression of the genes controlling pyruvate and acetoin production from glucose is downregulated under anaerobic conditions. In addition, the acetate production pathway only works aerobically, and pyruvate can be converted to acetate through acetyl-CoA. This conversion is carried out by the pyruvate dehydrogenase complex, encoded by *pdhABCD*. Notably, *pdhABCD* was downregulated under anaerobic growth conditions [7]. Therefore, the decreased levels of intracellular c-di-AMP under anaerobic conditions might be expected due to the lack of pyruvate. Contrary to this expectation, significantly higher levels of c-di-AMP concentrations were obtained under anaerobic conditions than under aerobic conditions (*p* < 0.05; Figure 6C and Figure 7B). Anaerobic conditions induce broad physiological changes in *L. monocytogenes*, including metabolic pathways, enhanced stress resistance, and increased virulence [79], which likely involves c-di-AMP signalling pathways. This also can be supported by our data showing that oxidative stress application led to a significant increase of c-di-AMP in both WT and Δ*pdeA* (*p* < 0.05; Figure 5).

## 5. Conclusions

In conclusion, our findings highlight the role of c-di-AMP in modulating catalase activity and cellular responses to oxidative stress. We showed that *pdeA* contributes to catalase activity levels of *L. monocytogenes*, however in this case, catalase activity does not play a significant role compared to c-di-AMP homeostasis in oxidative stress protection. The interruption in c-di-AMP homeostasis makes cells more sensitive towards oxidative stress in the early stationary phase. However, higher c-di-AMP accumulation renders Δ*pdeA* cells more robust compared to the early stationary phase. Our transcriptomic data explain that the sensitivity of Δ*pdeA* at 10 h is likely due to the downregulation of oxidative stress-related pathways, mainly PPP. Moreover, the robustness of Δ*pdeA* at 18 h was explained with a possible role in improved membrane functions or involvement of other pathways with further c-di-AMP accumulation. Although our transcriptomic data provides subtle changes in gene expression, it the c-di-AMP accumulation, might signify an effect of the above pathways. This suggests that c-di-AMP homeostasis might play a key role in various pathways and stress resistance. 1For the first time, we found that oxidative stress, supplementation with cysteine, and growth under anaerobic conditions elevated the c-di-AMP levels in *L. monocytogenes*. Therefore, our study is significant for understanding the bacterial response of *L. monocytogenes* and similar microorganisms to oxidative stress.

## Figures and Tables

**Table 1:** Strains used in this study.

Strain	Relevant Properties	Reference Source
10403S	Serotype ½ a, wild type, isolated from skin lesion	[36]
10403S Δ*pdeA*	In-frame deletion (knockout)	[37]

**Table 2:** The expression of the genes related to c-di-AMP homeostasis in *L. monocytogenes* 10403S WT cells grown in DM and 1.57 mM L-cysteine-containing DM overnight under anaerobic conditions.

Gene Name	Locus Tag	Log_2_fold Change	*p*-Value	*p-adj*	Gene Description
*Novel*	NI	1.12	1.14 × 10^−5^	1.58 × 10^−5^	cyclic-di-AMP receptor
*pdeA*	*lmo0052*	−0.77	5.11 × 10^−10^	8.54 × 10^−10^	cyclic-di-AMP phosphodiesterase
*pgpH*	*lmo1466*	−2.34	1.19 × 10^−139^	4.23 × 10^−138^	cyclic-di-AMP phosphodiesterase
*dacA*	*lmo2120*	−3.16	1.48 × 10^−109^	3.13 × 10^−108^	Diadenylate cyclase

NI: Not identified.

## Data Availability

The data generated and analysed during this study are available from the main author or corresponding author upon reasonable request.

## Supplementary Materials

The following supporting information can be downloaded at https://www.mdpi.com/article/10.3390/microorganisms13061400/s1. Table S1: Summary of RNA-seq alignment. Table S2: Transcription levels of genes playing a role in oxidative stress resistance (10 h grown Δ*pdeA* vs. WT in BHI under aerobic conditions).

## Abbreviations

The following abbreviations are used in this manuscript:

ATP	Adenosine Triphosphate
BHI	Brain Heart Infusion
BP	Biological Processes
c-di-AMP	Cyclic di-AMP
CC	Cellular Compound
DACs	Diadenylate Cyclases
DM	Defined Media
ESI	Electrospray Ionisation
GO	Gene Ontology
HPLC	High-Performance Liquid Chromatography
KEGG	Kyoto Encyclopedia of Genes and Genomes
LC-MS	Liquid Chromatography–Mass Spectrometry
MF	Molecular Function
PDEs	Phosphodiesterases
PPP	Pentose Phosphate Pathway
ROS	Reactive Oxygen Species
SOD	Superoxide Dismutase
TCA	Tricarboxylic Acid Cycle
WT	Wild Type
