# Supplementary material for: Impact of c-di-AMP Accumulation, L-cysteine, and Oxygen on Catalase Activity and Oxidative Stress Resistance of Listeria monocytogenes 10403S
Source: Microorganisms. 2025 Jun 16;13(6):1400. doi: 10.3390/microorganisms13061400 (PMC12196104; doi:10.3390/microorganisms13061400)
Supplement: Supplementary file 1 [file microorganisms-13-01400-s001.zip › microorganisms-3690188-supplementary.pdf]

Supplementary Materials:

Table S1: Summary of RNA-seq alignment.

Sample name	Raw reads	Clean reads	Raw bases	Clean bases	Error rate(%)	Q20(%)	Q30(%)	GC content(%)
Δ*pdeA*	18278272	17922976	2.7G	2.7G	0.01	99.18	97.58	40.94
Δ*pdeA*	12386370	11476508	1.9G	1.7G	0.01	98.15	94.97	44.59
Δ*pdeA*	15444426	15149490	2.3G	2.3G	0.01	99.21	97.62	40.01
WT	16674188	16310974	2.5G	2.4G	0.01	99.15	97.49	40.33
WT	12773682	12461640	1.9G	1.9G	0.01	98.61	95.84	39.83
WT	18495642	18179744	2.8G	2.7G	0.01	99.23	97.63	41.03

Table S2: Transcription levels of genes playing a role in oxidative stress resistance (10h grown Δ*pdeA* vs WT in BHI under aerobic conditions)

Gene Name	log_2FoldChange	*p* value	*padj*	Gene Description
cspA	-0.58573	0.599722	0.95182	Cold shock protein
cspB	-1.1015	0.296999	0.913794	Cold shock protein
cspD	0.181493	0.926222	0.987557	Cold shock protein
gshAB	0.515894	0.067853	0.827156	Glutathione biosynthesis bifunctional protein
katA	-0.28371	0.263357	0.913794	Catalase
ohrR	-0.06449	0.793181	0.974681	Organic hydroperoxide resistance transcriptional regulator
perR	0.09943	0.677095	0.955567	Peroxide operon regulator
prfA	-0.83698	0.019196	0.755653	Listeriolysin regulatory protein
rex	-0.29085	0.4971	0.948627	Redox-sensing transcriptional repressor
sigB	0.344826	0.33931	0.915327	RNA polymerase sigma-B factor
sodA	-0.43209	0.18168	0.890175	Superoxide dismutase
spxA	0.079911	0.783342	0.974681	Global transcriptional regulator
